# Low-grade appendiceal mucinous neoplasm and endometriosis of the appendix

**DOI:** 10.1186/s12957-017-1294-1

**Published:** 2017-12-19

**Authors:** Kyle D. Klingbeil, Basem Azab, Mecker G. Moller

**Affiliations:** 1 University of Miami Miller School of Medicine, 1475 NW 12th Ave, 3rd Flr, Miami, FL 33136 USA; 2 Department of Surgical Oncology, Sylvester Comprehensive Cancer Center, Miami, FL 33136 USA

**Keywords:** Low-grade appendiceal mucinous neoplasm, Endometriosis, Mucocele, Pseudomyxoma peritonei

## Abstract

**Background:**

A distended, mucous-filled appendix is known as an appendiceal mucocele. They are a rare form of an appendiceal mass and develop from both benign and malignant processes. Mucoceles can develop secondarily to an obstruction, such as from a fecalith, scarring or, rarely, endometriosis. Only 12 cases of non-neoplastic appendiceal mucoceles caused by endometriosis have been previously described. The association between neoplastic appendiceal mucoceles in the presence of endometriosis is described for the first time in this report.

**Case presentation:**

A 57-year-old woman presented with a chief complaint of worsening abdominal pain over the past 3 months. Imaging studies revealed an appendiceal mass. Laparoscopic evaluation confirmed an appendiceal mucocele, and the patient underwent complete appendectomy. No evidence of mucinous or endometrial deposits were present within the abdominal cavity. Pathological diagnosis revealed low-grade appendiceal mucinous neoplasm (LAMN) with evidence of endometriosis within the muscularis propria of the appendix. The patient recovered without complications and her abdominal pain completely resolved.

**Conclusions:**

Endometriosis of the appendix is a rare manifestation and is most often identified as an incidental finding. Endometriosis leading to an obstructive mucocele of the appendix is an exceedingly rare finding, having only been described 12 times in the medical literature. LAMN in the presence of endometriosis of the appendix is described for the first time in this report. The association between appendiceal neoplasms in the presence of endometriosis requires further research in order to optimize operative treatment.

## Background

Appendiceal mucoceles are defined as a distended, mucous-filled appendix. It is a rare cause of an appendiceal mass, found in approximately 0.3% of appendectomy specimens [1, 2]. Mucoceles are further classified by histological subtypes. Non-neoplastic mucoceles include mucosal hyperplasia and simple cysts. Neoplastic mucoceles include mucinous adenoma, low-grade appendiceal mucinous neoplasm (LAMN), and appendiceal adenocarcinoma, as defined by the World Health Organization (WHO) [3]. A minority of mucoceles can also develop secondary to obstruction (e.g., fecaliths, scarring, cecal neoplasms) leading to retention of mucous and resultant appendiceal distention. Mucoceles are typically found incidentally during radiographic or endoscopic evaluation. Surgical resection is almost always indicated due to the underlying risk for malignancy and perforation, which can lead to pseudomyxoma peritonei (PMP).

Other causes of an appendiceal mass include acute appendicitis, appendiceal neoplasms (e.g., leiomyoma, fibroma, lipoma, carcinoid, adenocarcinoma), duplication cysts, tuberculosis, and endometriosis. Appendiceal endometriosis is a rare finding, with a prevalence of 2.8% in patients with known endometriosis and in approximately 0.3% of appendectomy specimens [4, 5].

An appendiceal mucocele in the presence of endometriosis is an extremely rare finding, having only been previously described 12 times in the literature. However, in all of the previous cases, the mucocele was found to be caused secondarily to the overt growth of endometriosis leading to obstruction. Herein, we discuss a case of an appendiceal mucocele revealed to be a LAMN with an incidental finding of endometriosis within the muscularis propria of the appendix, findings which have not been previously described. We include a review of the current literature to expand on the pathophysiology and management of this disease entity.

## Case presentation

**Fig. 1:**
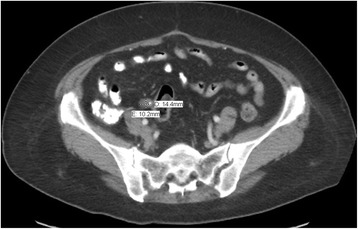
A transverse section of an abdominal CT with PO and IV contrast, revealing a mass obstructing the distal end of the appendix, measuring 14.4 mm × 10.2 mm

A 57-year-old Nicaraguan woman initially presented to her primary care physician with a 3-month history of diffuse lower abdominal pain associated with abdominal fullness. Her symptoms were thought to be secondary to constipation, and the patient was treated conservatively. The pain progressively worsened over the next couple of weeks, localizing to the right lower quadrant (RLQ) with associated episodes of nausea and vomiting. The patient then presented to the emergency department with severe abdominal pain. Computed tomography (CT) imaging revealed a 14.4 × 10.2 mm mass at the tip of the appendix without evidence of acute appendicitis (Fig. 1).

Past medical history was significant for a total abdominal hysterectomy and bilateral salpingo-oophorectomy for symptomatic endometriosis, completed in Nicaragua 16 years earlier. She denied taking hormone replacement therapy and denied episodic flushing, diarrhea, or wheezing. On physical examination, there were no palpable abdominal masses, and the RLQ was mildly tender, but without rebound or guarding.

**Fig. 2:**
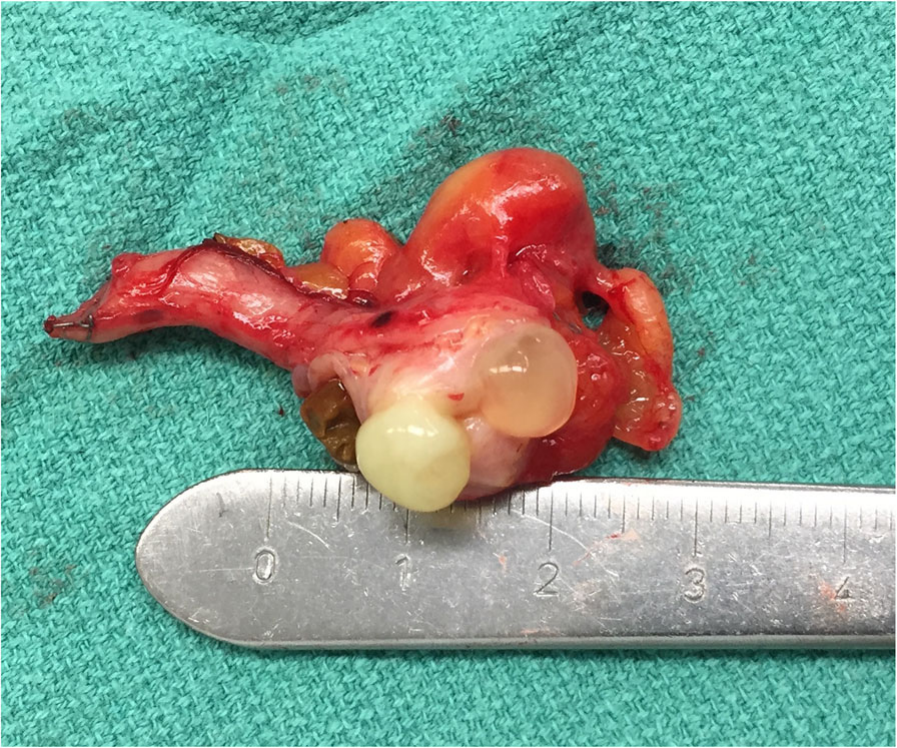
Gross appearance of appendiceal mass in cross section, notable for distention of the appendiceal lumen with no evidence of herniation or perforation through the appendiceal wall, measuring 1.5 cm in greatest dimension

**Fig. 3:**
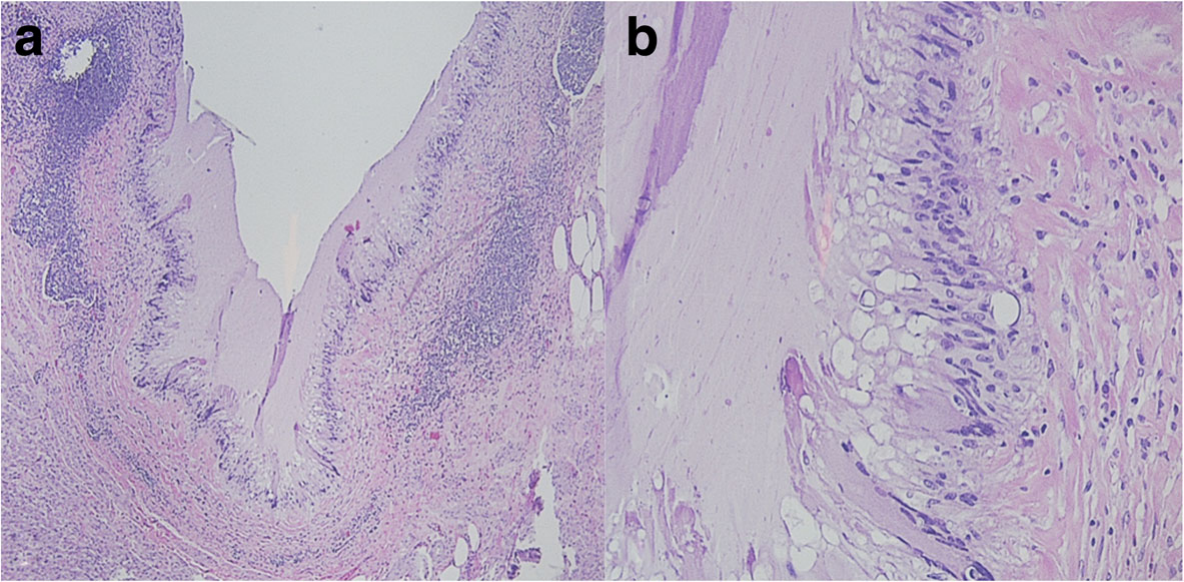
**a** Appendiceal mass biopsy at low power (×40 magnification) and **b** high power (×200 magnification) of the low-grade appendiceal mucinous neoplasm, exhibiting crowded columnar epithelial cells with elongated, hyper-chromatic nuclei and excessive apical mucin (hematoxylin and eosin stain). There is no evidence of invasion into the submucosa

**Fig. 4:**
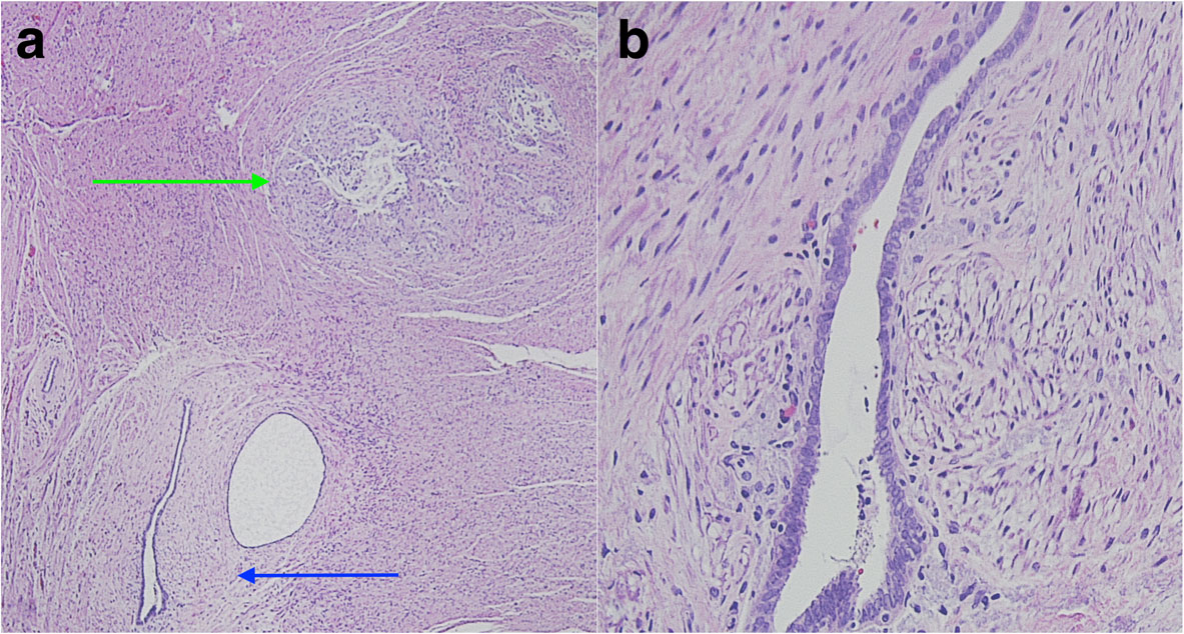
**a** Appendiceal mass biopsy at low power (×40 magnification) showing evidence of the low-grade appendiceal mucinous neoplasm (marked by green arrow) and endometriosis (marked by blue arrow). **b** High power view (×200 magnification) of an endometrial gland, exhibiting simple cuboidal epithelium surrounded by endometrial stromal cells with spindle appearance (hematoxylin and eosin stain)

**Fig. 5:**
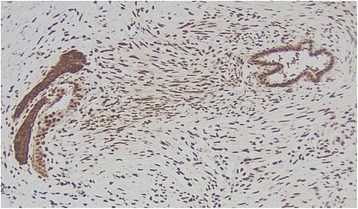
Appendiceal mass biopsy at medium power (×100 magnification) showing concentrated expression of estrogen receptors within endometrial glands and more diffusely in surrounding endometrial stroma (estrogen receptor alpha antibody [SP1] immunohistochemistry stain)

A preoperative colonoscopy revealed no gross involvement of the cecum or base of the appendix. The patient then underwent surgical resection using a laparoscopic approach. Upon initial inspection of the intra-abdominal contents, no gross evidence of endometriosis was visualized; however, moderate adhesions were present within the lower midline pelvis. The appendix was visualized, appearing without evidence of appendicitis, and was resected at the base of the cecum. The gross specimen appeared ill-defined and soft, measuring 1.5 cm in largest diameter. A cross-section of the mass showed a white-tan inner surface with areas of mucin-filled cysts (Fig. 2). Pathology demonstrated LAMN without evidence of adenocarcinoma, and negative proximal margins (Fig. 3). Within the muscularis propria of the appendiceal wall, evidence of endometriosis was incidentally discovered (Fig. 4), exhibiting positive estrogen receptor (ER) expression (Fig. 5). The patient tolerated the surgery well and recovered without any major complications.

## Discussion

Of the various types of neoplastic appendiceal mucoceles, LAMN, previously known as appendiceal neoplasm of uncertain malignant potential, refers to a tumor with neoplastic adenomatous growth, in the presence of low-grade cytologic atypia and other secondary defining factors (Table 1) [6, 7]. These neoplasms are known to herniate and perforate through the appendiceal wall, with potential to cause PMP, even after complete resection [8].

**Table 1:** Classification of epithelial neoplasia of the appendix

Terminology	Defining factors	Secondary defining factors
Adenoma	Tubulor, tubulovillous, or villous adenoma with low- to high-grade dysplasia	
Low-grade appendiceal mucinous adenoma	Mucinous neoplasm with low-grade cytologic atypia	Presence of ≥ 1 of the following:
		-Submucosa fibrosis
		-Loss of muscularis mucosae
		-Diverticulum-like growth through appendiceal wall
		-Dissection of acellular mucin
		-Mucin outside the appendix
		-Appendiceal rupture
Mucinous adenocarcinoma	Mucinous neoplasm with evidence of cellular invasion past the epithelial layer	

Further classification of LAMN has been defined in attempts to stratify the risk of PMP. LAMN type I is described as a non-distended appendix with proliferation of mucinous epithelium, or a distended appendiceal lumen in the presence of dysplastic mucinous epithelium. LAMN type II is described as having mucin herniating into the appendiceal wall, perforating through the appendiceal wall or the presence of extra-appendiceal mucin along the outer surface of the appendix. Patients with type II LAMN have been shown to have a higher risk of PMP compared to those with type I LAMN [9]. This case in particular appears to be most compatible with type I LAMN, confined to the appendiceal lumen as confirmed by pathologic evaluation.

The management of LAMN, like other appendiceal mucoceles, is primarily based on surgical intervention. Early diagnosis and resection is important due to the risk of underlying malignancy and risk of perforation. Cases of appendiceal masses that appear benign by imaging studies have been shown to have underlying cystadenocarcinoma [10]. Therefore, standard appendectomy should be considered for all appendiceal masses, regardless of their appearance. In cases with a homogenous mass of the appendix, without nodularity or evidence of distant spread, a laparoscopic approach is suggested. The laparoscopic approach must include stapling through the base of the cecum to avoid spreading neoplastic cells or mucin into the peritoneum. Complicated mucoceles involving the cecum or terminal ileum and cystadenocarcinomas with adjacent organ involvement require more extensive resection, often resulting in a right hemicolectomy.

PMP is a rare complication of abdominal mucinous tumors and is characterized by the dissemination of peritoneal mucinous deposits, with the potential to progress to severe ascites. The majority of PMP is derived from appendiceal tumors [9]. LAMN can therefore be viewed as a precursor to PMP. Appropriate surgical management is thus an important aspect for both the physician and patient. In the presence of PMP, urgent cytoreductive surgery followed by hyperthermic intraperitoneal chemotherapy (HIPEC) has been found to be the most fundamental surgical option, notable for a significant increase in disease-free survival rates [11]. The classification system developed by McDonald et al. further stratifies patients with PMP based upon LAMN pathologic features. Patients with LAMN type I require management by watchful waiting only, whereas those with LAMN type II necessitate cytoreductive surgery and HIPEC [9].

Another important aspect of appendiceal mucocele management includes the evaluation for concurrent malignancies. Appendiceal mucoceles are associated with tumors of the gastrointestinal tract, ovary, endometrium, and breast [12–14]. Colorectal carcinoma can be found in approximately 20% of patients; therefore, a preoperative or intraoperative colonoscopy is often indicated [15]. Colonoscopic evaluation is also advantageous in determining whether or not an appendiceal mass extends into the cecum and/or terminal ileum, guiding appropriate surgical management.

The presence of endometriosis in an appendiceal mucocele is a rare finding. It is diagnosed pathologically, expressing glandular tissue with endometrial stroma and hemorrhage, localizing most commonly to the muscularis propria and serosal layers of the appendix [16]. Hapke and Bigelow first described the possible mechanism between endometriosis and the development of an appendiceal mucocele in a step-by-step process: endometriosis leads to the progression of smooth muscle hypertrophy in the muscularis propria resulting in obstruction of adjacent gland crypts. This obstruction leads to the entrapment of mucin production generating mucinous cysts that eventually coalesce. As the cysts grow, they begin to herniate and eventually perforate through the submucosal and serosal layers of the appendix [17].

At present, it remains unclear whether or not the presence of endometriosis influences the neoplastic potential of an appendiceal mucocele. However, malignancies have been shown to arise from endometrial deposits and the presence of endometriosis increases the risk for other malignancies, particularly ovarian carcinoma [18]. Therefore, it is possible that endometriosis may affect the local tissue environment of an appendiceal mucocele, increasing the risk for neoplastic transformation.

A review of the literature revealed 13 total cases of appendiceal mucocele in the presence of endometriosis, including the present case, summarized in (Table 2) [5, 17, 19–27]. Of the 13 female patients, the average age was 38.8 years old and the most common presenting symptom was chronic abdominal pain. Only two cases had a known history of endometriosis, the rest being diagnosed upon surgical exploration. Complications included two cases of appendiceal intussusception, one case of ureteral obstruction, one case of appendiceal tethering to an ovarian cyst and one case of appendiceal rupture. Upon pathological investigation, all previous cases demonstrated a simple mucocele with evidence of endometriosis involving the muscularis propria and serosa leading to luminal obstruction. Operative management varied depending on the involvement of the endometrial implantation. The most common surgical approach was an open ileocecal resection (*n* = 5), followed by open appendectomy (*n* = 4). No cases of recurrence or malignant transformation were reported.

**Table 2:** Summary from a review of the current literature involving appendiceal mucocele and endometriosis, involving a total of 12 studies and 13 cases

Author, year	*N*, age	History of Endometriosis?	Clinical presentation	Diagnostic evaluation	Pathological diagnosis	Location of appendiceal endometriosis	Operative management
Abrao, 2005	1, 32	No	CAP	US	SM	MPS	ICR
Akagi, 2008	1, 35	No	Anemia	Colonoscopy	SM, AI	MPS	LA, PC
Driman, 2000	2,	#1 No	#1 CAP	#1 Colonoscopy	#1 SM	#1 MPS	#1 RH, OC
	#1 34	#2 No	#2 CAP and infertility	#2 DL	#2 SM	#2 MPS	#2 LA
	#2 31						
Hapke, 1977	1, 31	No	Dysmenorrhea	EL	SM	MPS	EL, OA
Kimura, 1999	1, 41	No	AAP and vomiting	EL	SM, AI	MPS	EL, OA
Klingbeil, 2017	1, 57	Yes	CAP	CT, Colonoscopy	LAMN	MPS	LA
Kohout, 1960	1, 44	No	Menorrhagia	Clinical	SM	MPS	TAH, OA
Miyakura, 2012	1, 56	No	Positive FOBT	Colonoscopy, CT, MRI	SM, Ruptured	MPS	ICR
Nopajaroonsri, 1994	1, 22	No	AAP and vomiting	Clinical	SM	MPS	OA
O’Sullivan, 2001	1, 31	No	Right flank pain	Urogram, US, CT	SM	MPS	ICR
Shemilt, 1949	1, 47	Yes	AAP	Clinical	SM	MPS	ICR
Tsuda, 2013	1, 43	No	Dysmenorrhea	CT, MRI, Colonoscopy	SM	MPS	ICR

*AAP* acute abdominal pain, *AI* appendiceal intussusception, *CAP* chronic abdominal pain, *CT* computed tomography, *DL* diagnostic laparoscopy, *EL* exploratory laparotomy, *FOBT* fecal occult blood test, *ICR* ileocecal resection, *LA* laparoscopic appendectomy, *LAMN* low-grade appendiceal mucinous neoplasm, *MPSA* muscularis propria and serosa, *MRI* magnetic resonance imaging, *OC* ovarian cystectomy, *OA* open appendectomy, *PC* partial cecectomy, *RH* right hemicolectomy, *SM* simple mucocele, *TAH* total abdominal hysterectomy, *US* ultrasound

## Conclusion

Endometriosis of the appendix is a rare manifestation and is most often identified as an incidental finding. Endometriosis leading to an obstructive mucocele of the appendix is an exceedingly rare finding, having only been described 12 times in the medical literature. LAMN in the presence of endometriosis of the appendix is described for the first time in this report.

The appropriate preoperative evaluation and surgical management for patients with an obstructive appendiceal mucocele is of utmost importance due to the underlying risk for a neoplastic etiology, e.g., LAMN, and its associated complication of PMP. The diagnosis of LAMN should therefore be viewed as a precursor to PMP. Patients who present with PMP may necessitate cytoreductive surgery and HIPEC, based on the recent classification system developed by McDonald et al. 2012.

Herein, an appendiceal mass was diagnosed as LAMN with an incidental finding of endometriosis. It is currently uncertain whether or not the presence of endometriosis influences the neoplastic potential of an appendiceal mucocele. Further investigation is required in order to guide surgical management of this rare disease entity.

## Abbreviations

CT: Computed tomography
ER: Estrogen receptor
HIPEC: Hyperthermic intraperitoneal chemotherapy
LAMN: Low-grade appendiceal mucinous neoplasm
PMP: Pseudomyxoma peritonei
RLQ: Right lower quadrant
WHO: World Health Organization
